# A Comparison of Self-Perceived Oral and Facial Esthetics in Patients After Lip Repositioning Surgery With Modified and Conventional Techniques

**DOI:** 10.7759/cureus.50206

**Published:** 2023-12-08

**Authors:** Reham Al Jasser

**Affiliations:** 1 Periodontics and Community Dentistry, King Saud University, Riyadh, SAU

**Keywords:** facial appearance, gummy smile, lip surgery, lip repositioning surgery, gingival display, orofacial esthetics

## Abstract

Background: Patients with excessive gingival display (EGD) are treated with lip repositioning surgeries (LRS). This study used a questionnaire to analyze and evaluate how patients who received LRS with modified and traditional techniques perceived their own oral and facial esthetics at various timelines after their surgeries.

Methods: An orofacial esthetic questionnaire (OEQ) was used in this cross-sectional study. The participants were patients who underwent LRS for the treatment of their EGD. They were divided into control (n=100) and test (n=100) groups. For the control group, LRS were performed using traditional/conventional techniques, and for the test group, LRS were performed using a modified approach. An OEQ was used to record responses. The scale comprised eight questions targeted to capture participating patients' perceptions about their own oral and facial esthetics at four timelines (baseline and one-month, six-month, and one-year follow-up). Patients responded to each question on a 10-point Likert scale (0: very dissatisfied, 10: very satisfied). Data was analyzed by independent samples T-tests using Statistical Package for the Social Sciences (SPSS) version 21.0 software (IBM SPSS Statistics, Armonk, NY).

Results: Seven (3.5%) out of the total (N=200) patients were unable to take part in the study's OEQ. At one-year follow-up, the mean and standard deviation (SD) for the test group's gingival display (GD) were 2.48±0.86 mm and 3.77±1.76 mm, respectively, and comparisons revealed that the test group's GD was significantly lower (p=0.000) than the control group. Results from the participant responses to OEQ using the Likert scale at one-year follow-up revealed significant differences between the control and test groups for all questions, except question 5 (p=0.06), as the shape of the teeth will not be affected by LRS. Patients in the test group who underwent LRS with a modified approach have a high level of satisfaction (satisfaction score: >9). Patients in the control group scored their satisfaction less favorably, with certain questions (question 3) receiving scores as low as 0.31. Perception of oral and facial esthetics was significantly higher for the test group at different time points. At one-year follow-up, the mean difference was 4.46, which was the greatest (p=0.000).

Conclusions: EGD improved significantly at one year with the modified lip repositioning technique. The satisfaction level of patients with outcomes of the modified lip repositioning was significantly higher as compared to the satisfaction level of patients who underwent the conventional technique.

## Introduction

The symmetry of the face, the shape of the nose, the eyes, the teeth, and the tissues around the teeth, such as the gums, lips, and cheeks, have historically been seen to be the most significant aspects [1]. Dental appearance involves the gingival display (GD) and location of the upper lip in addition to the color, size, shape, and placement of the teeth. Therefore, despite having a healthy dentition, excessive gingival display (EGD) can often damage and worsen a patient's oral and facial esthetics [2].

The health of adolescents and young adults, particularly females, is significantly impacted by oral and facial esthetics, which affects how they see their bodies and their sense of self [3]. Oral characteristics have a significant influence on these young people's biopsychosocial behavior [4]. Orofacial characteristics have a considerable effect on how people portray themselves, how their oral health is affected, and how they interact with others on a social level [3,4]. This is especially true for women. In contrast to those with oral diseases, who display a poor body image and a higher level of isolation, females with superior orofacial esthetics are viewed as more attractive and have a better social influence [5]. It has been asserted that facial features, particularly oral esthetics, have the potential to affect how young females perceive themselves, particularly during the period of life when there is a lot of social and affective interaction, despite the lack of reliable evidence that this can have a long-term negative impact on psychosocial well-being [6].

Physical beauty is a significant social relationship component for young women. Thus, esthetic changes to the face may be recognized by oneself and may have an impact on one's quality of life [7]. For instance, among young adults in Finland, improving dental appearance and attitudes about malocclusion were the main drivers for orthodontic treatment [8]. Teenagers in a Brazilian research who had undergone orthodontic treatment reported better oral health outcomes when they could smile, laugh, and display their teeth without feeling self-conscious [9].

As an esthetic smile becomes a more important part of what it means to be beautiful, more people are being encouraged to seek corrective and cosmetic operations. The attractiveness and esthetics of a smile depend on a number of factors [10]. Esthetic perception can vary depending on cultural, socioeconomic, environmental, and personal factors such as experience and educational level [11]. Previous research has indicated that people find a smile to be more attractive when there is less gingival display (GD), with dental professionals being more critical of gingival presentation than laypeople [12]. The optimal GD is between 1 and 3 mm, according to research by numerous authors. While there are numerous factors that can affect how appealing a smile seems, excessive GD (EGD), also known as a gummy smile, is one of the primary problems associated with an unsatisfactory dental smile and is regarded as a crucial element in smile analysis [13]. EGD is defined as a full, vivacious smile with an excess of more than 2-4 mm of gingival show. This may be more noticeable if the lips are hypermobile. At least 50% of patients have some form of GD in a typical smile. Up to 76% of all patients, nevertheless, could exhibit exaggerated or forced smile patterns [14,15]. The gingival margin of the anterior central incisors and the inferior border of the upper lip should be separated by 1-2 mm in a "normal" smile. In contrast, an excessive gingiva-to-lip distance of 4 mm or more is seen as "unattractive" by both laypeople and general dentists [16,17].

Gingival enlargements, bony maxillary excess, insufficient maxillary lip length, hypermobile upper lip, and excessive bone in the maxilla are just a few of the possible etiologies for EGD. The therapeutic approach should therefore be centered on the main etiology or the combination of etiologies found in each case [13-17]. During a dynamic smile, the upper lip should move somewhere between 4 and 6 mm from rest. Lip translation from the relaxed posture to the largest smile position, used in clinical examination, can detect hypermobile upper lips [18]. One treatment option to address this excessive translation is lip repositioning surgery (LRS), a predictable surgical procedure [17]. To start improving a patient's smile, dental professionals must first accurately diagnose the etiology before undertaking any kind of treatment. Lip repositioning surgery is a cosmetic operation that reduces the elevator pull muscles, which lift the upper lip, to remedy a gummy smile [18]. To maintain the top lip near your teeth and reduce the amount of exposed gum, the surgery limits how high the upper lip can rise when you smile. Under local anesthetic, the procedure entails making two incisions beneath the point where your gums and upper lip converge. Between these two incisions, a flap of gum tissue is excised, and the upper and lower parts are stitched together [19]. Only 3-4 days are normally required for recovery time. LRS often has a very low risk of infection, bleeding, and pain compared to other surgical procedures [20]. Bruising, swelling, and soreness can occur in some people, but these adverse effects are typically mild. Scars are typically concealed in the mouth. Few case papers have recently discussed the modified LRS procedure, although several have recently documented the standard LRS technique for treating EGD [13-20].

The perception of facial and dental esthetics has been studied using a variety of methods [21]. Patients receiving orthodontic or prosthodontic treatment frequently report their perceptions of orofacial attractiveness using the orofacial esthetic questionnaire (OEQ) [22]. The OEQ is utilized in the current study to investigate and assess how patients who underwent LRS with traditional and modified techniques felt about their own oral and facial esthetics at different points after their surgery. The null hypothesis asserts that there are no variations between the patients' perceptions of their face and oral esthetics at the various postoperative intervals.

## Materials and methods

Ethical approval

The Institutional Review Board (IRB) and Institutional Committee of Research Ethics of King Saud University in Riyadh, Saudi Arabia, approved the study for ethical considerations (permission number: E-18-113207). The 2013 revision of the 1975 Helsinki Declaration was followed while conducting the study. Prior to their enrolment, all participants provided their informed permission.

Selection and calculation of sample sizes

The G Power software determined the final sample size of 200 patients with a confidence level of 95% and a moderate effect size, with 100 patients in each of the control and test groups.

Subjects, study design, and setting

A sample of 200 young Saudi female adult patients who wished to have LRS to reduce their excessive gingival presentation were solicited to participate in the study and were subsequently recruited from July 2014 to April 2016. The present study is an extension of the previously published randomized controlled trial [17].

The communication was tailored to each participant after selecting an appropriate target population for the study and included all pertinent information regarding the measures and associated clinical parameters of the suggested surgical procedures, as well as the study's objectives, design, risks, and potential benefits. The principal investigator made contact with the study's intended audience and requested their participation.

**Figure 1:**
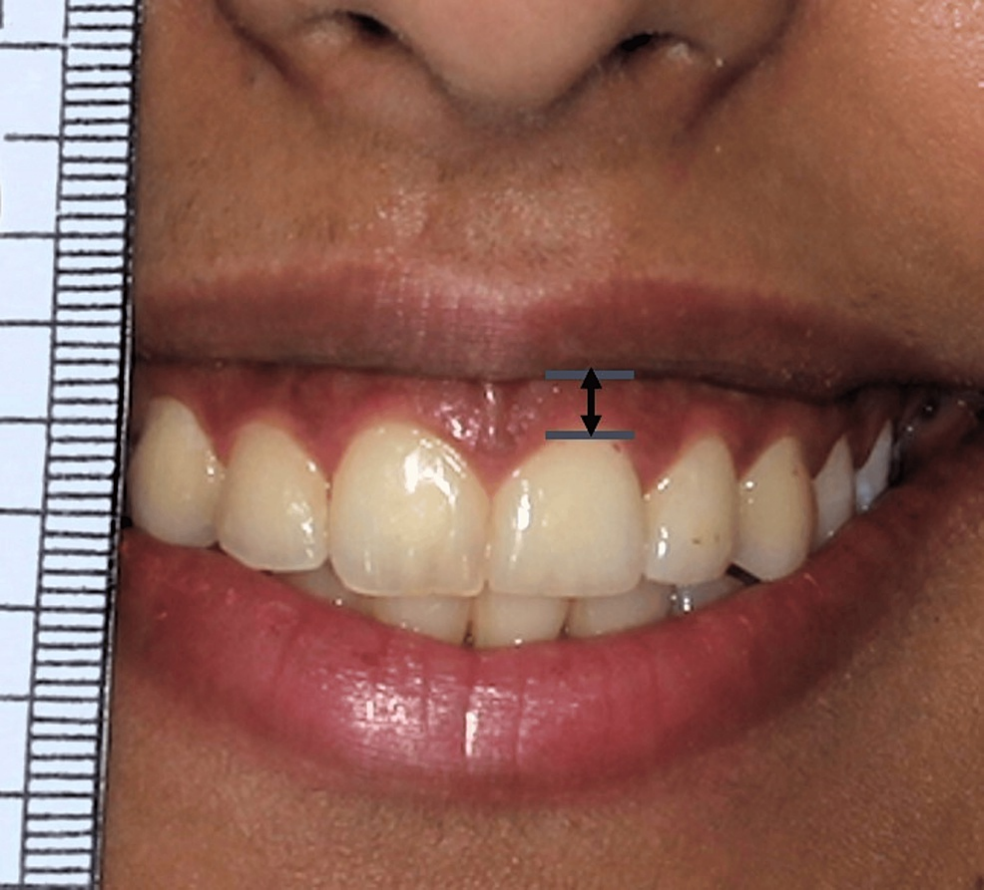
Pictorial presentation of the measurement of the gingival display using a ruler in millimeters The arrow denotes the measurement of the gingival display from the lip line to the free gingival margin.

The consenting participants were split into two equal groups (control and test) at random. Patients in the control group underwent LRS using the traditional/conventional procedure, while patients who participated in the test group underwent LRS utilizing the modified approach. The primary distinction is that, in modified LRS, the mucosa is preserved and a submucosal tunnel is created instead of a strip of mucosa being removed from the maxillary vestibule and the lip mucosa sutured to the mucogingival junction. The lead investigator (RNA) performed all surgical procedures and examinations for calibration [17]. While the patient was smiling the widest, the gingival display above the right central incisor of the maxilla was measured in millimeters (Figure 1).

Lip repositioning surgery performed for the control group

**Figure 2:**
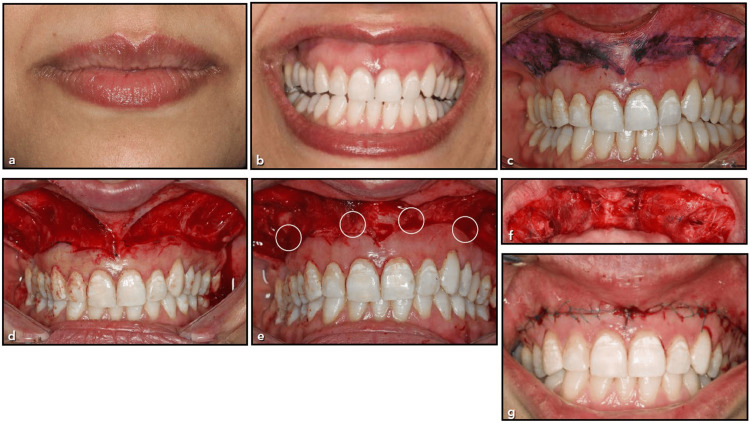
(a-g) Surgical procedure in the test group (a and b) Presurgical assessment of the ALW, VLT, and AGD. (c and d) Mucosa (15 mm) was bilaterally removed apical to the mucogingival junction. (e and f) Four periosteal vertical simple interrupted sutures were placed to hold thick connective tissue fibers in a more apical direction. (g) Continuous interlocking sutures obtained proper closure of the surgical site. ALW: average lip width at rest, VLT: vertical lip translation with maximum smile, AGD: average gingival display

The surgical approach employed by this group used the LipStaT® method as described by Bhola et al. [23]. The boundaries of the area of the surgical incision were marked out with a surgical marker. The inferior border extended to the first molar area bilaterally and was 1 mm coronal to the mucogingival junction based on the dynamic smile's horizontal expansion. The height of the superior incision within the vestibule was estimated at 15 mm based on a 2:1 ratio of vertical extension being twice the measurement of EGD at a complete dynamic smile. Using scalpel blade number 15, superior and inferior incisions were made, and they were connected bilaterally by two vertical incisions. The indicated mucosa strip was removed using a partial thickness dissection, revealing the connective tissue fascia underneath. Next, using continuous interlocking polypropylene 4/0 sutures (PROLENE® Polypropylene Suture, Ethicon US, LLC, Cincinnati, OH) that began on one side of the incision and finished on the other, the surgical site was appropriately closed. This suture anchored the newly formed gingiva mucosal border in its new location (Figure 2c, 2d, 2g) [24].

Modified lip repositioning surgery performed for the test group

**Figure 3:**
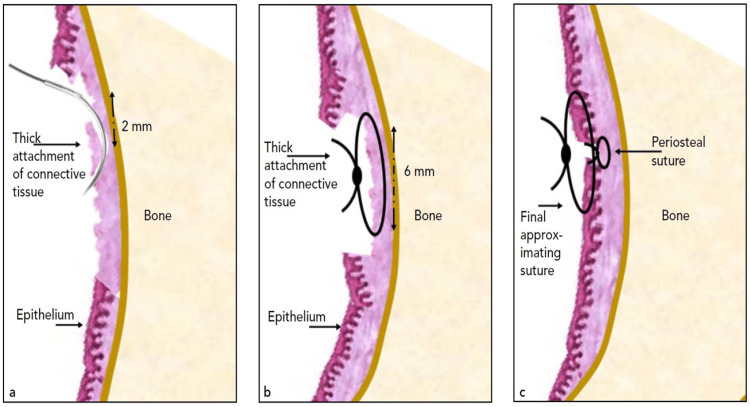
Schematic drawings portraying the periosteal suturing utilized (a) The needle is inserted, starting 2 mm coronal to the base of the thick connective tissue attachment or frenal attachment, and then, the needle is slid apically, passing the attachment. (b) Sliding the needle up to 6 mm and tying a knot creates a simple interrupted suture. (c) The suture is intended to move and stabilize the thick connective tissue attachments in a more coronal position.

The test group received the identical surgical technique, but before the continuous interlocking sutures, a periosteal simple interrupted suture was placed [24]. In areas where there are strong frenal attachments or connective tissue, this vertical simple interrupted suture was used. Before tying the knot, the needle was positioned by starting it 2 mm coronal to the base of the connection and moving it apically by crossing the connective tissue attachment up to 6 mm. This suture was intended to shift and stabilize the thick connective tissue attachments into a more coronal position. For all periosteal sutures, Vicryl 4-0 resorbable sutures (Vicryl Rapide^TM^ (polyglactin 910) Suture, Ethicon US, LLC, Cincinnati, OH) were utilized. Usually, 3-4 periosteal sutures were used per surgical site (Figure 2 and Figure 3) [24].

Follow-up visits

**Figure 4:**
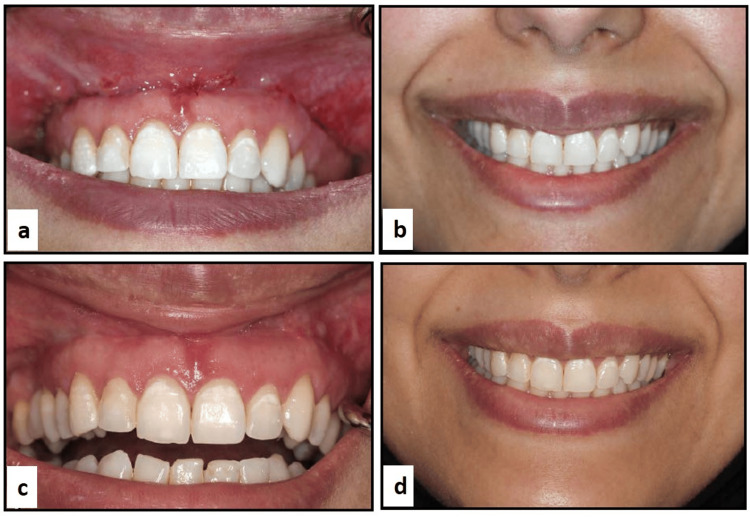
(a-d) Follow-up visits (a and b) Follow-up at one week post-surgery. (c and d) Follow-up at six months post-surgery.

Following surgery, each participant received weekly appointments for the first four weeks, then appointments at one month, six months, and one year (Figure 4) [24].

Orofacial esthetic questionnaire (OEQ)

The answers of the subjects were recorded using the OEQ (Table 1) [25,26]. The scale, which is of eight questions, was designed to measure participants' opinions of their own facial and oral esthetics at four time points.

**Table 1:** Orofacial esthetic scale used to record the responses* of the participants *Responses were evaluated based on a Likert scale: 0: very dissatisfied and 10: very satisfied.

Questions	
Q1	Your facial appearance
Q2	Appearance of your facial profile
Q3	Your mouth appearance (smile, lips, and visible teeth)
Q4	Appearance of your rows of teeth
Q5	Shape/form of your teeth
Q6	Color of your teeth
Q7	Your gum appearance
Q8	Overall, how do you feel about the appearance of your face, mouth, and teeth?

The questionnaire termed OEQ evaluated the orofacial esthetics. Its reliability and validity were assessed while it was being developed for prosthodontic and orthodontic patients. There are eight questions in this instrument. Participants are asked about how they feel about the way their face, mouth, teeth, and artificial teeth look. The participants gave their feedback on a scale of 0-10 (with 0 denoting "very dissatisfied" and 10 denoting "very satisfied"), or if they did not want to give feedback, they checked the "not applicable" box. Seven esthetic elements were mentioned in the OEQ items (face, facial profile, mouth, teeth in rows, tooth shape/form, tooth color, and gum). The summative score for these seven components ranged from 0 to 70 (the maximum score was achieved when the patient was entirely satisfied). An eighth OEQ item described how the patient felt about the orofacial esthetics overall.

Follow-up appointments

The following follow-up appointments were scheduled: baseline (before the procedure), one month after the procedure, six months after the procedure, and one year after the procedure. At each of the many follow-up appointments after the LRS, OEQ were given to the participants. Patients answered each of the eight questions on a Likert scale with a maximum score of 10, where a higher score indicates better esthetics (0: very dissatisfied, 10: very satisfied).

Data analysis

The summary results of the OEQ questions and participant sociodemographic data were examined for central tendency (means) and variability (standard deviation (SD)). Student T-tests were employed in the Statistical Package for the Social Sciences (SPSS) version 21.0 software (IBM SPSS Statistics, Armonk, NY) to make sure that both test and control groups were compared and could be evaluated for statistically significant differences. The independent samples T-test was used to compare the gingival display between the test and control groups at various dates. In 193 (96.5%) subjects, all eight questions on the orofacial esthetic scale were answered. Only seven (3.5%) participants did not participate and did not finish the survey, so they were not included in the analysis of the survey items.

## Results

The GD data of all 200 participating patients were documented. However, seven (3.5%) of the total patients were unable to take part in the study's orofacial esthetic evaluation questionnaire portion and record their responses.

Table 2 compares the means of the GD measured in millimeters (mm) for the control and test groups of the involved patients at four different time points. Nonsignificant differences (p>0.05) were seen between the control and test groups at the baseline, one-month, and six-month measurements. The GD for the control (3.77±1.76 mm) and test (2.48±0.86 mm) groups, however, exhibited a significant difference at one-year follow-up (p=0.000).

**Table 2:** Comparison of gingival display at different timelines for the test and control groups with independent samples T-test (N=200) *P-value was significant at p<0.05.

Gingival display at different timelines	Group	Mean	Standard deviation	Standard error of the mean	Mean difference	*Significance (two-tailed)
Baseline	Control	5.27	0.75	0.075	-0.09	0.409
	Test	5.36	0.87	0.087		
1 month	Control	2.35	0.84	0.084	0.07	0.500
	Test	2.27	0.75	0.075		
6 month	Control	2.39	0.82	0.082	0.16	0.146
	Test	2.23	0.77	0.077		
1 year	Control	3.77	1.76	0.176	1.28	0.000
	Test	2.48	0.86	0.086		

**Figure 5:**
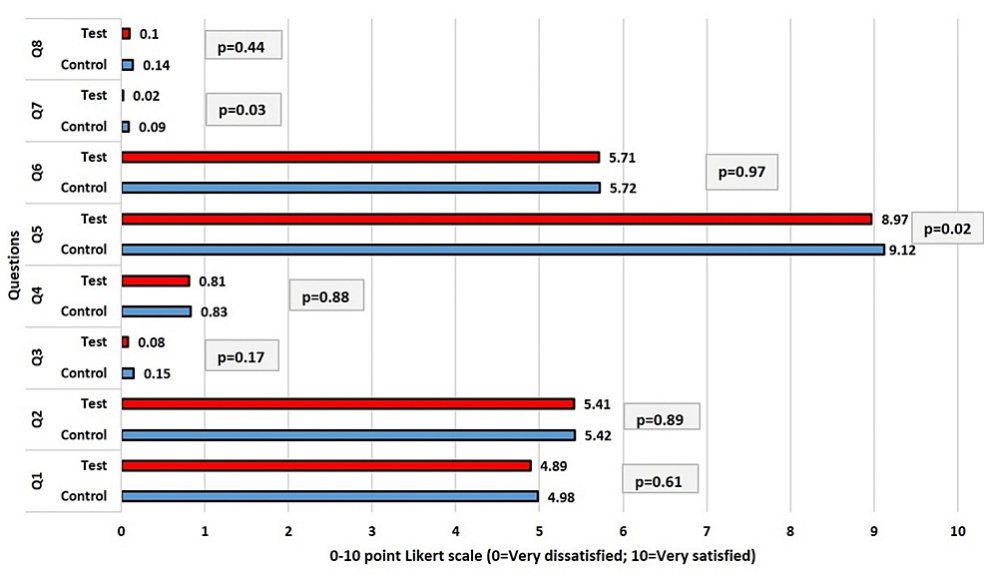
Comparison of responses (Q1-Q8) of the participants at baseline

The baseline data for participant responses to the eight questions of the OEQ are shown in Figure 5 on a Likert scale. Patients in the control and test groups responded in very identical ways. It was clear from the participants' responses that nearly all of them were unhappy with the appearance of their mouths (smile, lips, and visible teeth) (question 3), the appearance of their teeth (question 4), the appearance of their gums (question 7), and the overall appearance of their mouth, gums, and teeth (question 8).

**Figure 6:**
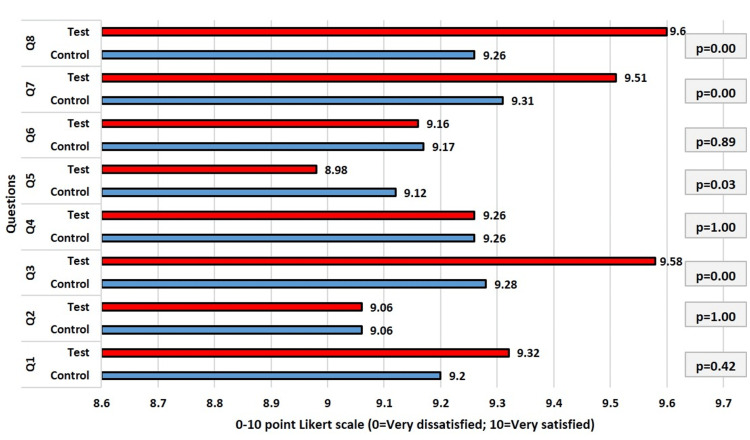
Comparison of responses (Q1-Q8) of the participants after one month

The statistics from the participant replies to the eight questions of the OEQ at one-month follow-up are shown in Figure 6. Regarding the participants' ratings of the responses given by the patients in the control and test groups using the Likert scaling set, they were practically identical. Although comparisons for questions 3, 5, 7, and 8 revealed statistical differences (p<0.05), the largest difference in response values was just 0.34 between the control (9.26) and test (9.6) group responses. Overall, the test and control group participants' replies indicated a significant increase in patients' levels of satisfaction compared to the data gathered at the baseline.

**Figure 7:**
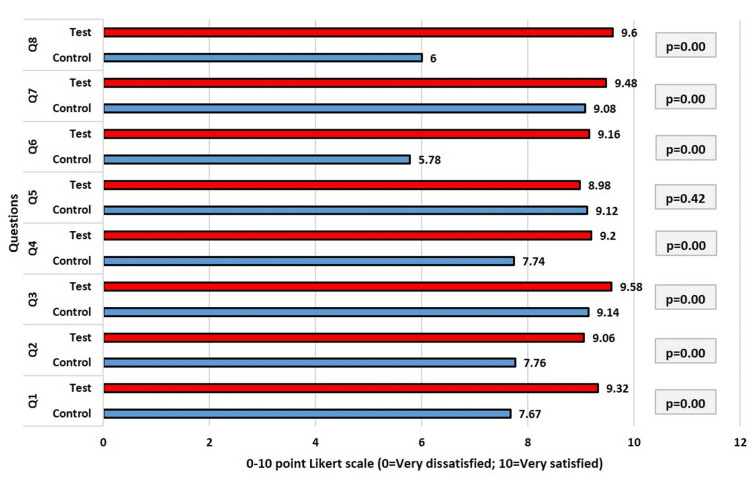
Comparison of responses (Q1-Q8) of the participants after six months

Figure 7 presents the results from the participant responses to the eight questions of the OEQ recorded using the Likert scale at six-month follow-up. With the exception of question 5 (p=0.42), the values of the patient replies in the control and test groups indicated significant differences (p<0.05) for all of the questions at six-month follow-up. The test group and control group's replies varied most (3.6) and least (0.14), respectively, for questions 8 and 5, respectively. Overall, the total replies from the test group maintained a higher level of patient satisfaction when compared to the total replies from the control group, according to the values of the responses provided by the test and control group members.

**Figure 8:**
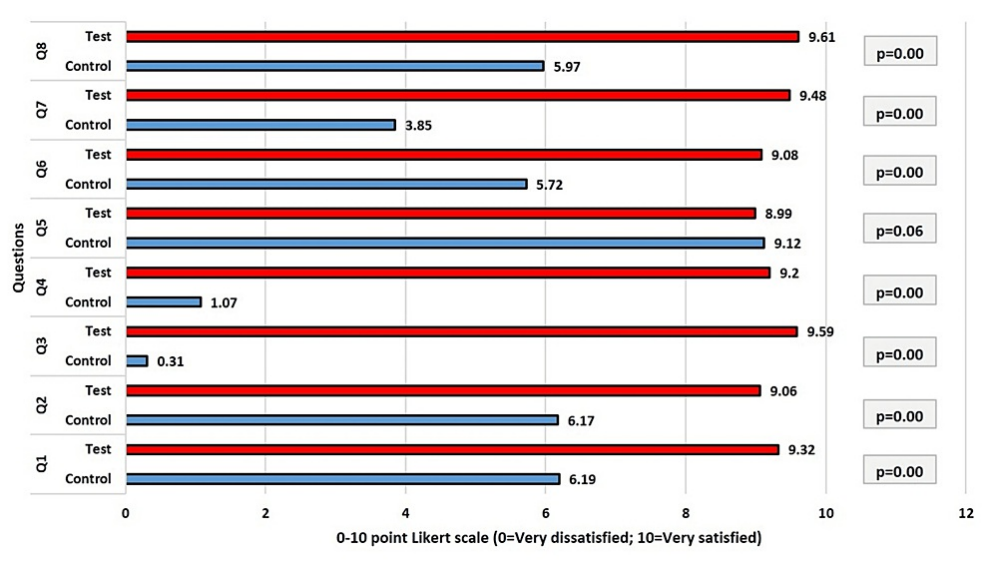
Comparison of responses (Q1-Q8) of the participants after one year

Figure 8 shows the results from the participant responses to the eight questions of the OEQ evaluation recorded using the Likert scale at one-year follow-up. There were significant differences between the patient response values in the control and test groups at one-year follow-up for all questions, with the exception of question 5 (p=0.06), which is understandable given that the surgical technique used in the study will not affect the shape of the teeth (question 5). Participants' replies revealed that patients in the test group category who underwent LRS with the modified approach were more satisfied. The test group's participating patients' average satisfaction score was higher than 9, indicating a high level of satisfaction. Patients in the control group scored their satisfaction less favorably, with certain questions (question 3) receiving scores as low as 0.31.

Table 3 compares the overall responses (Q1-Q8) of the test and control groups at various times using an independent samples T-test. Perception of oral and facial esthetics, as judged by the study participants, was found to be significantly higher for the test group than for the control group at different time points. At one-year follow-up, the mean difference was 4.46, which was the greatest (p=0.000). This proved that patients in the test group who received LRS with the modified approach had greater long-term patient satisfaction levels than those in the control group who received LRS using the standard technique.

**Table 3:** Comparison of overall responses (Q1-Q8) at different timelines for the test and control groups with independent samples T-test *P-value was significant at p<0.05.

Overall mean of responses (Q1-Q8)	Group	Mean	Standard deviation	Standard error of the mean	Mean difference	*Significance (two-tailed)
Baseline	Control	3.28	0.50	0.050	0.05	0.428
	Test	3.22	0.49	0.049		
1 month	Control	9.20	0.30	0.030	-0.10	0.008
	Test	9.30	0.21	0.021		
6 month	Control	7.78	0.41	0.041	-1.51	0.000
	Test	9.29	0.22	0.022		
1 year	Control	4.74	0.68	0.068	-4.46	0.000
	Test	9.21	0.95	0.095		

## Discussion

The OEQ can be used to measure orofacial esthetics, which is a dimension of oral health-related quality of life, a comprehensive and significant notion to characterize how people view their oral health [22-26]. The present study expands the scope of the scale's original use for prosthodontic or orthodontics patients by evaluating the orofacial esthetics of periodontal patients who underwent LRS to treat an excessively gummy smile.

The success of the majority of dental procedures performed on patients includes not only restoring the stomatognathic system's lost functions but also enhancing the face's attractiveness and harmony with the rest of the body [27]. Research and clinical observations revealed that patients' perceptions of facial esthetic characteristics frequently differed from professionals' perceptions [28]. Nevertheless, it is difficult to quantify because it depends on a number of variables, including age, gender, educational attainment, culture, and measurement method [27,28]. The patient's satisfaction with orofacial esthetic appearance and the impact of its impairment, influencing the patient's psychosocial quality of life, are used to assess the patient's self-perception of orofacial appearance [21-28]. Orofacial esthetics evaluations can be performed at many points in time, including before and after dental work has been done [25]. The OEQ was utilized in this study as the study tool to assess how the participating patients felt about their orofacial look. The successful use of OEQ in previous studies with similar objectives has been reported in the literature [22-30]. To assess the overall satisfaction of the participating patients with their esthetic appearance prior to LRS, after one month, after six months, and after one year following the surgery, the patients' satisfaction levels regarding their orofacial condition were targeted in all the questions asked.

When time and resources are limited, the OEQ is a quick and easy-to-use questionnaire that can be used to assess the face and oral esthetics of dental patients [29]. The OEQ is a practical and time-saving tool for epidemiological studies, national health surveys, and ordinary dentistry practice where a multi-item OEQ questionnaire is not viable due to a larger patient sample size [30]. This is because of its concision and simplicity of use. In the past, sophisticated multi-item surveys were employed more frequently than single-item surveys, which were thought to be less accurate, valid, and comprehensive [29,30]. Currently, this pattern is changing among practitioner researchers, who now favor the use of single-item questionnaires, particularly in clinical settings, because they take less time, are easier for respondents to complete, and are less expensive to collect data from [31]. Multi-item instruments may be more discriminating and better suited for complex constructs, but they can take longer and may result in answer errors. This offers an opportunity that is both conceptually appealing and practical and may be used in extensive epidemiological studies, clinical trials, and ordinary dental care, all of which would enhance the field of evidence-based dental practice [31-33].

The chances of relapse are high as time progresses after LRS. This is a normal relapse post-LRS according to the literature as the upper lip muscle attachment will tend to go back to its original place with continuous dynamic movement of lips after surgical detachment [14-18]. The results of the present study demonstrated advancements in the clinical characteristics of excessive gingival display in the test group by demonstrating lower scores than the control group for the assessed gingival display at various time points. The gingival display at one-year follow-up was excellent, demonstrating that there had been no relapse in the test group, and it was much lower than that of the control group. According to the responses that the participating patients recorded, the psychological impact was likewise strong, with the participants in the test group expressing significantly higher levels of satisfaction than the participants in the control group. According to the findings of the current study, the results of lip repositioning surgery with a modified method were stable even after a year of follow-up, and the participants who underwent this surgery were pleased with the results. Thus, the null hypothesis of no variations between the patients' perceptions of their face and oral esthetics at the various postoperative intervals with the two lip repositioning techniques was rejected. The modified lip repositioning surgery employed in this study can therefore be considered less invasive and more conservative and can be adopted for the treatment of excessive gingival display, which is also supported by prior literature [14-17]. This is in comparison to other treatment modalities for excessive gingival display [13,34,35].

Numerous practitioners experimented with a variety of methods to treat their patients' annoying gummy smile issues, including lip repositioning, cosmetic crown lengthening, gingival depigmentation, a combined strategy for a gummy smile makeover and micro-autologous fat transplantation, orthognathic surgery that necessitates general anesthesia, and aggressive bony osteosynthesis [34-36]. In comparison to orthognathic surgery, lip repositioning surgery offers a cautious and less aggressive surgical approach and has shown predictive results in the treatment of EGD [14-17]. The current study discovered that one year following the modified lip repositioning operation, LRS could result in an overall EGD reduction of 2.88 mm. These findings are somewhat in line with earlier research that showed an average decline of 2.71-3.4 mm [35] at six months and 2.10 mm [37,38] at a year. However, the unique aspect of this study was how it contrasted the standard lip repositioning approach with the previously unreported modified technique. The two crucial findings of the current investigation are the direct comparison of the EGD at various time points following the two surgical methods and the considerable improvement of EGD with the modified LRS.

Study limitations

Within the constraints of this investigation and based on the overall evidence derived from the findings, LRS is more effective at treating EGD while also generating greater patient satisfaction levels. Due to the gender selection, the results of this study should be evaluated with care. Female patients make up the majority of the research group, which is consistent with several studies in the field of cosmetic dentistry because women are more conscious of their oral and facial looks than men are, and they also place greater importance on esthetics [5]. Young people made up the majority of the patients who participated and received LRS for the current study in terms of average age. The esthetic requirements of people at this age and the fact that the EGD condition tends to gradually improve with age because of decreasing muscle tone resulting in lower lip mobility may be related to this [39]. To obtain more substantial and definitive results regarding the outcome, long-term stability, and the patient's response and happiness with the improved LRS approach, additional carefully planned clinical trials are nonetheless required.

## Conclusions

Within the limitations of the current study, it can be concluded that excessive gingival display improved significantly at one-year follow-up with the modified lip repositioning surgical technique as compared to the conventional/classic lip repositioning technique. According to the participating patients' responses, the satisfaction level of patients with outcomes of the modified lip repositioning surgery was significantly higher as compared to the satisfaction level of patients who underwent lip repositioning surgery with the conventional technique.

The orofacial esthetic scale used in the study is a promising instrument for the assessment of orofacial esthetics and can be used in future studies with similar objectives.
